# Cardiac complications in autosomal dominant polycystic kidney disease: links to genotype and CKD severity

**DOI:** 10.1093/ckj/sfaf279

**Published:** 2025-10-01

**Authors:** Giulia Condello, Pierluigi Fulignati, Viola D’Ambrosio, Erica Rosati, Alessandra Terracciano, Chiara Tacente, Ilaria Mariani, Luca Calvaruso, Giuseppe Grandaliano, Francesco Pesce

**Affiliations:** Nephrology, Dialysis and Transplantation Unit, Department of Medical and Surgical Sciences, Fondazione Policlinico Universitario A. Gemelli IRCCS, Rome Italy; Nephrology, Dialysis and Transplantation Unit, Department of Medical and Surgical Sciences, Fondazione Policlinico Universitario A. Gemelli IRCCS, Rome Italy; Nephrology, Dialysis and Transplantation Unit, Department of Medical and Surgical Sciences, Fondazione Policlinico Universitario A. Gemelli IRCCS, Rome Italy; Nephrology, Dialysis and Transplantation Unit, Department of Medical and Surgical Sciences, Fondazione Policlinico Universitario A. Gemelli IRCCS, Rome Italy; Translational Cytogenomics Research Unit, Bambino Gesù Children’s Hospital, IRCCS, Rome, Italy; Nephrology, Dialysis and Transplantation Unit, Department of Medical and Surgical Sciences, Fondazione Policlinico Universitario A. Gemelli IRCCS, Rome Italy; Università Cattolica del Sacro Cuore, Rome, Italy; Nephrology, Dialysis and Transplantation Unit, Department of Medical and Surgical Sciences, Fondazione Policlinico Universitario A. Gemelli IRCCS, Rome Italy; Università Cattolica del Sacro Cuore, Rome, Italy; Nephrology, Dialysis and Transplantation Unit, Department of Medical and Surgical Sciences, Fondazione Policlinico Universitario A. Gemelli IRCCS, Rome Italy; Nephrology, Dialysis and Transplantation Unit, Department of Medical and Surgical Sciences, Fondazione Policlinico Universitario A. Gemelli IRCCS, Rome Italy; Università Cattolica del Sacro Cuore, Rome, Italy; Università Cattolica del Sacro Cuore, Rome, Italy; Division of Renal Medicine, Ospedale Isola Tiberina-Gemelli Isola, Rome, Italy

**Keywords:** ADPKD, left ventricular hypertrophy, *PKD1*, *PKD2*, valvular heart disease

## Abstract

**Background:**

Autosomal dominant polycystic kidney disease (ADPKD), caused by *PKD1/PKD2* mutations, features renal and extrarenal manifestations including valvulopathies and left ventricular hypertrophy (LVH), which increase mortality. Associations between these cardiac abnormalities and the ADPKD genotype or disease severity remain poorly defined. We investigated the prevalence and associations of valvulopathies and LVH with renal function, renal size, systemic features and genotype in ADPKD.

**Methods:**

This retrospective, single-centre study analysed 154 adult ADPKD patients. Data included echocardiography (LVH, valvulopathies), abdominal ultrasound (renal diameter), cranial magnetic resonance imaging, estimated glomerular filtration rate (eGFR) and genetic testing (*PKD1/PKD2* mutations) in 87 patients. Associations were assessed using appropriate statistical tests including logistic regression for multivariable analysis.

**Results:**

Aortic regurgitation was associated with larger mean renal diameter (*P* = .027) and lower eGFR (*P* < .001). However, when adjusted for gender and age the associations are no longer significant. Interventricular septal thickness correlated positively with renal diameter (*r* = +0.32, *P* < .001) and negatively with eGFR (*r* = −0.39, *P* < .001). Left ventricular hypertrophy (LVH, prevalence 30%) was significantly associated with *PKD1* mutations (*PKD1* non-truncating versus *PKD2* adjusted *P*-value = .011; *PKD1* truncating versus *PKD2* adjusted *P*-value = .011), independent of hypertension, age, sex and anaemia (adjusted OR 8.5, *P* = .008). Mitral valve prolapse was associated with truncating *PKD1* mutations (*P* = .007), independent of hypertension, age, sex and anaemia (adjusted OR 3.950, *P* = .037). No associations were found with hepatic, pancreatic or intracranial cysts/aneurysms.

**Conclusions:**

This study demonstrates an independent association between LVH and *PKD1* mutations and links aortic regurgitation with renal disease severity in ADPKD. These findings highlight genotype–phenotype correlations that may help stratify cardiovascular risk and inform personalized management in ADPKD.

KEY LEARNING POINTS **What was known:**

- Autosomal dominant polycystic kidney disease (ADPKD) causes significant cardiovascular morbidity/mortality, including left ventricular hypertrophy (LVH) and valvulopathies.
- Increased prevalence of LVH and valvular issues in ADPKD were recognized but often attributed mainly to hypertension.
- Clear associations between specific cardiac abnormalities and ADPKD genotype (*PKD1* versus *PKD2*) or disease severity markers were largely lacking.

**This study adds:**

- A significant association exists between LVH and *PKD1* mutations, independent of hypertension and other confounders, suggesting direct genetic influence.
- A novel link is identified between aortic regurgitation and markers of ADPKD severity (larger renal diameter, lower estimated glomerular filtration rate).
- An association between mitral valve prolapse and truncating *PKD1* mutations is confirmed, reinforcing genotype-specific cardiac phenotypes.

**Potential impact:**

- Identifying *PKD1* mutation carriers may flag patients at higher intrinsic risk for LVH, prompting earlier or more intensive cardiovascular surveillance and management.
- The link between aortic regurgitation and renal disease severity could refine risk stratification, highlighting patients needing closer cardiac monitoring as kidney function declines.
- These findings support genotype-informed personalized medicine in ADPKD, potentially guiding tailored screening and therapeutic strategies for cardiovascular complications.

## INTRODUCTION

Autosomal dominant polycystic kidney disease (ADPKD) is the most common monogenic [1] inherited nephropathy, caused by mutations in either the *PKD1* or *PKD2* gene, which encode polycystin-1 and polycystin-2, respectively. In addition to renal involvement, ADPKD is associated with extrarenal manifestations, including cysts in other organs [2, 3], intracranial aneurysms [4], abdominal wall hernias [5], colonic diverticula [6] and cardiovascular abnormalities [7] such as hypertension, valvulopathies and left ventricular hypertrophy (LVH). Cardiovascular complications represent the leading cause of death in ADPKD patients [8]. The systemic expression of polycystins raises questions about their direct role in cardiovascular pathophysiology beyond the effects of hypertension.

Valvulopathies and LVH increase the risk of developing further cardiovascular disorders, including arrhythmias, systolic/diastolic dysfunction and ischaemic heart disease. However, data on the association between these cardiac abnormalities and the ADPKD phenotype/genotype remain limited. The literature describes a higher prevalence of LVH in ADPKD patients during childhood [9], adolescence [10] and adulthood [11, 12] compared with the general population. However, it remains controversial whether this increased prevalence is solely secondary to hypertension or if intrinsic factors related to ADPKD pathogenesis contribute directly. Similarly, previous retrospective studies on valvulopathies in ADPKD have primarily confirmed their higher prevalence compared with the general population [12–14]. However, no study has yet demonstrated an association between heart valve disease and the renal or systemic features of ADPKD. Of particular interest, a recent study by Miyamoto *et al.* [15] documented an association between *PKD1* mutations and mitral regurgitation in a cohort of 65 ADPKD patients.

The objectives of this study were to assess the prevalence of LVH and valvular disease (specifically aortic, mitral, pulmonary and tricuspid regurgitation, as well as mitral prolapse) in a large cohort of ADPKD patients and to investigate potential

associations between these echocardiographic abnormalities and renal diameter, renal function, systemic manifestations (liver, pancreatic and intracranial cysts and intracranial aneurysms) and genotype. Understanding the underlying pathogenetic mechanisms may aid in identifying at-risk patient subgroups and developing personalized interventions.

## MATERIALS AND METHODS

### Study design

This observational, retrospective study was conducted at the Agostino Gemelli Hospital in Rome on a single-centre cohort of 154 ADPKD patients. The inclusion criteria were age ≥18 years and an ultrasound-confirmed diagnosis of ADPKD based on the unified Pei–Ravine criteria (including a documented positive family history in cases where it was required for diagnostic confirmation) [16]. The only exclusion criterion was the initiation of renal replacement therapy during the study period. All included patients had previously undergone transthoracic echocardiography, abdominal ultrasound, cranial magnetic resonance imaging (MRI)/angio-MRI and blood chemistry tests between January 2018 and October 2024.

Genetic testing was performed in 87 patients, categorized into three subgroups: *PKD1* truncating mutations, *PKD1* non-truncating mutations and *PKD2* mutations. The estimated glomerular filtration rate (eGFR) used for statistical analysis was obtained at the time of echocardiography and was calculated using the Chronic Kidney Disease Epidemiology Collaboration formula. Hypertension was assessed based on patient history and medical records, defined according to European Society of Cardiology/European Society of Hypertension guidelines [17] as blood pressure (BP) ≥140/90 mmHg or treatment with antihypertensive medications (diuretics, calcium channel blockers, renin–angiotensin–aldosterone system (RAAS) inhibitors or beta-blockers).

The study was conducted in accordance with the ethical principles outlined in the Declaration of Helsinki. The study protocol and procedures, including those related to patient informed consent and data protection, were reviewed and approved by the local ethics committee (Comitato Etico Territoriale Lazio Area 3, Rome, Italy; approval ID: 6290).

### Echocardiographic assessment

Transthoracic echocardiography was performed to evaluate LVH and valvular abnormalities. LVH was assessed using two-dimensional measurement of the interventricular septum thickness, with a cut-off value of 11 mm, in accordance with the European Association of Cardiovascular Imaging and American Society of Echocardiography (ASE) guidelines [18]. Valvular abnormalities, including regurgitation of the mitral, aortic, pulmonary or tricuspid valves, as well as mitral valve prolapse, were assessed using two-dimensional echocardiography and Doppler imaging. The severity of valvular regurgitation was classified as mild, moderate or severe based on ASE guidelines [19].

### Abdominal ultrasound

Abdominal ultrasound was used to confirm the diagnosis of ADPKD according to the Pei–Ravine unified criteria. Ultrasound-measured renal length is a validated surrogate for height-adjusted total kidney volume and disease progression in ADPKD, showing excellent correlation with MRI-derived volumes (*r* ≈ 0.9) [20, 21]. Renal diameter was assessed as the maximum longitudinal length and averaged for both kidneys. In addition, the presence of hepatic and pancreatic cysts was assessed in all participants.

### Cranial MRI

Cranial MRI was used to assess intracranial cysts (including those affecting the pineal gland, arachnoid membranes and choroid plexus) and intracranial aneurysms. Cranial magnetic resonance angiography was performed only in patients with an eGFR >30 ml/min/1.73 m^2^ due to the potential risk of nephrogenic systemic fibrosis associated with contrast media. In patients with an eGFR ≤30 ml/min/1.73 m^2^, cranial MRI without contrast was performed.

### Genetic analysis

Genetic testing was performed in a subset of 87 patients using next-generation sequencing (NGS). In cases where NGS did not detect disease-causing mutations, multiplex ligation-dependent probe amplification was used to improve the detection of copy number variations. This combined approach provided a comprehensive genetic analysis in all tested patients. Across the guanine–cytosine (GC)-rich exon 1 of *PKD1*, the panel achieved a mean read depth of 118× with ≥20× coverage for 92% of targeted bases; any stretches falling below this threshold (eight samples) were resequenced by Sanger using primers PKD1-E1-F/R to ensure complete coverage. Pathogenicity of all *PKD1*/*PKD2* variants was assessed according to the American College of Medical Genetics and Genomics/Association for Molecular Pathology 2015 guidelines using the VarSome platform, with evidence drawn from gnomAD version 2.1.1 (non-Finnish European subset; benign threshold minor allele frequency >0.0001), in-silico predictions (SIFT, PolyPhen-2, MutationTaster, CADD; SpliceAI for intronic variants) and published segregation or ClinVar/PKDB reports (see Supplementary Methods).

### Statistical analysis

Statistical analysis was conducted using SPSS 23 Statistics (IBM, Armonk, NY, USA). Categorical variables were reported as absolute numbers and percentages while continuous variables were summarized as mean ± standard deviation (SD). Renal diameter was calculated as the average of bilateral renal measurements. The *t*-test was used to compare renal diameter and eGFR between patients with and without valvulopathies. Comparisons were adjusted for gender and age by utilizing multivariate models. Pearson’s correlation test was applied to assess relationships between interventricular septal thickness and renal parameters (renal diameter and eGFR). The pairwise Fisher’s exact test was used to evaluate associations between genotype and echocardiographic features. Variability and differences in interventricular septal thickness among genotype subgroups was assessed using analysis of variance and Tukey’s Honest Significant Difference (HSD) post hoc test. The associations of LVH and mitral valve prolapse with *PKD1* mutations were adjusted for hypertension, age, sex and anaemia using logistic regression. A *P*-values <.05 were considered statistically significant for all analyses.

## RESULTS

### Clinical, laboratory and genetic characteristics

A total of 154 patients were included in this study, with a subset of 87 patients undergoing genetic testing. Among those genotyped, 53 patients (61%) had *PKD1* mutations, with 31 carrying truncating mutations and 22 carrying non-truncating mutations. The remaining 34 patients (39%) had *PKD2* mutations. Among the *PKD1* gene variants, 18 were classified as pathogenic, 26 as probably pathogenic and 10 as variants of uncertain significance (VUS; one individual was found to be a carrier of two *PKD1* gene mutations: one classified as probably pathogenic and the other as VUS). In the *PKD2* mutation group, 26 variants were classified as pathogenic, 6 as probably pathogenic and 2 as VUS (Supplementary Table 1). At the time of echocardiography, 103 patients (67%) were hypertensive. Table 1 summarizes the clinical, laboratory and genetic characteristics of the study cohort (see Supplementary Tables).

**Table 1:** Clinical, laboratory and genetic characteristics of the cohort.

Characteristics	Values	
Patients, *n*	154
Age (years), mean ± SD	48 ± 13
Sex (male), *n* (%)	78 (51)
eGFR (ml/min/1.73 m^2^), average ± SD	48 ± 13
CKD stage, *n* (%)	
I	32 (21)
II	43 (28)
IIIa	22 (14)
IIIb	30 (19)
IV	21 (14)
V	6 (4)
Intracranial aneurism, *n* (%)	9 (6)
Intracranial cysts, *n* (%)	11 (7)
Pancreatic cysts, *n* (%)	15 (10)
Liver cysts, *n* (%)	146 (95)
Arterial hypertension, *n* (%)	103 (67)
Subgroup with known genotype, *n*	87
*PKD2, n* (%)	34 (39)
*PKD1, n* (%)	53 (61)
Truncating/non-truncating	31/ 22

### Prevalence of valvulopathies and LVH

The prevalence of valvulopathies and LVH in the total study population is presented in Table 2. Mitral regurgitation was the most frequent valvular abnormality, observed in 104 patients (67%), although only 5 patients (4.8%) were classified as moderate and none were severe. Tricuspid regurgitation was detected in 83 patients (54%), with 3 patients (3.6%) classified as moderate. All cases of aortic and pulmonary regurgitation were mild. LVH was identified in 46 patients (30%).

**Table 2:** Prevalence of valvulopathies and LVH in the total population.

Echocardiographic features, *n* (%)	Patients (*N* = 154)
Mitral regurgitation	104 (67)
Mitral prolapse	35 (23)
Aortic regurgitation	31 (20)
Tricuspid regurgitation	83 (54)
Pulmonary regurgitation	9 (6)
LVH	46 (30)

### Association between cardiac abnormalities and ADPKD phenotype

We assessed the association of valvulopathies and LVH with renal diameter, renal function (eGFR) and systemic manifestations (hepatic, pancreatic, intracranial cysts and intracranial aneurysms).

**Figure 1:**
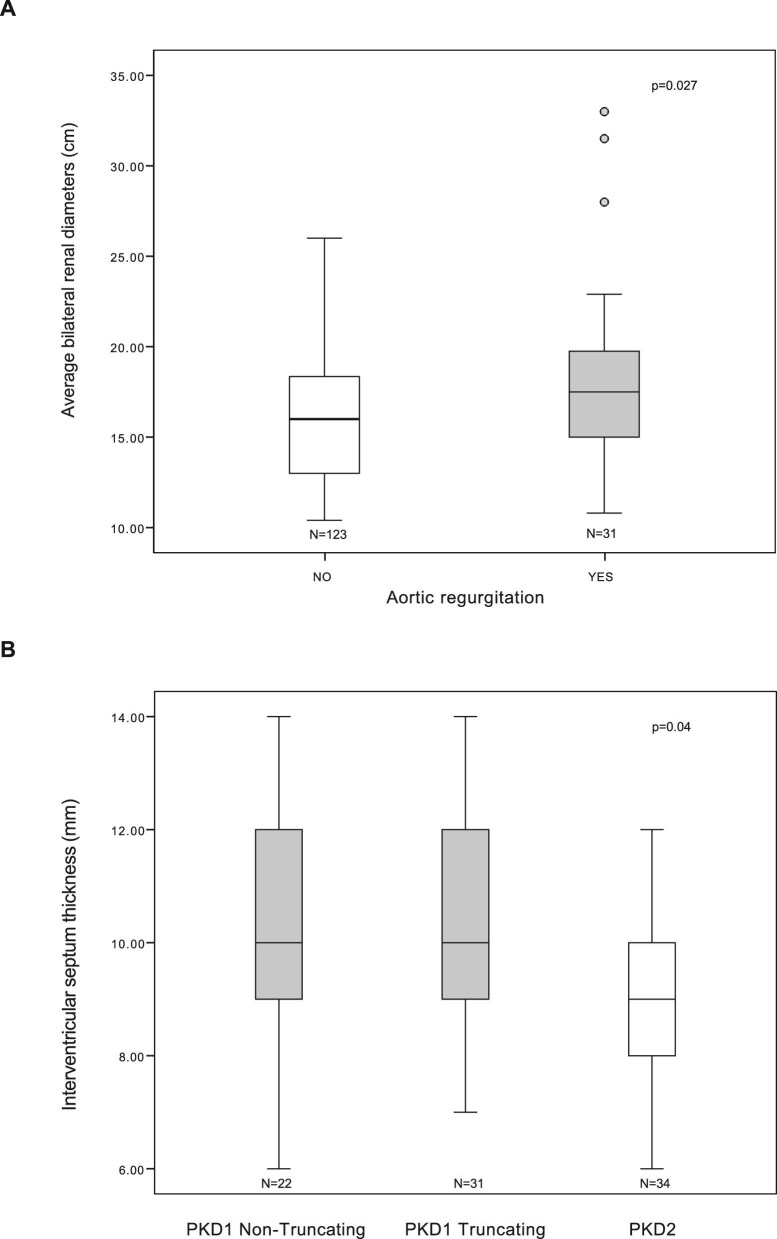
Associations between cardiac findings and clinical or genetic parameters in ADPKD patients. **(A)** Box plot comparing average bilateral renal diameter (cm) between patients with (Yes) and without (No) aortic regurgitation (*P* = .027, independent samples *t*-test). **(B)** Box plot comparing interventricular septum thickness (mm) among patients with *PKD1* non-truncating, *PKD1* truncating and *PKD2* mutations (*P* = .04, analysis of variance). Boxes represent interquartile range (IQR), horizontal lines indicate median, whiskers extend to 1.5 times the IQR and outliers are shown as circles.

Patients with aortic regurgitation had significantly larger renal diameters than those without this valvular abnormality (*P* = .027; Fig. 1A). The average bilateral renal diameter in patients with aortic regurgitation was 17.9 ± 5.4 cm, compared with 16.1 ± 3.7 cm in those without. Additionally, eGFR was significantly lower in patients with aortic regurgitation (40.9 ± 22.1 ml/min/1.73 m^2^) compared with those without (64.7 ± 31.5 ml/min/1.73 m^2^, *P* < .001). However, when adjusted with multivariate models for sex and age, the associations are no longer significant (Supplementary Table 2).

Interventricular septal thickness showed a positive correlation with renal diameter (*r* = +0.32, *P* < .001) and a negative correlation with eGFR (*r* = −0.39, *P* < .001).

Finally, no significant association was found between valvulopathies or LVH and the presence of hepatic, pancreatic or intracranial cysts or intracranial aneurysms (Supplementary Table 3). These findings suggest that cardiac abnormalities in ADPKD occur independent of other systemic manifestations (see Supplementary Tables).

### Association between cardiac abnormalities and genotype

Among the 87 genotyped patients, a significant association was found between mitral valve prolapse and *PKD1* truncating mutations (pairwise Fisher’s exact test *PKD1* truncating versus *PKD2* adjusted *P*-value = .007, Supplementary Table S4). The association remained statistically significant after adjustment for hypertension, age, sex and anaemia [*P* = .037, odds ratio (OR) 3.950; Supplementary Table 5). Among the 20 patients with mitral valve prolapse, 13 (65%) had *PKD1* truncating mutations, 5 (25%) had *PKD1* non-truncating mutations and 2 (10%) had *PKD2* mutations (Table 3). In *PKD1* patients, the interventricular septum thickness was significantly greater (Fig. 1B) than in *PKD2* patients (Tukey’s HSD post-hoc test *PKD1* non-truncating versus *PKD2* adjusted *P*-value = 0.034; *PKD1* truncating versus *PKD2* adjusted *P*-value =  0.017; see Supplementary Table 6). The average interventricular septum thickness was 10.4 ± 2.1 mm in the non-truncating *PKD1* subgroup, 10.5 ± 1.9 mm in the truncating *PKD1* subgroup and 9.1 ± 1.4 mm in the *PKD 2* subgroup (Fig. 1B). Additionally, LVH was significantly associated with *PKD1* mutations (pairwise Fisher’s exact test *PKD1* non-truncating versus *PKD2* adjusted *P*-value = 0.011; *PKD1* truncating versus *PKD2* adjusted *P*-value = 0.011), with no significant difference between truncating and non-truncating variants (Supplementary Table 7). The association remained statistically significant after adjustment for hypertension, age, sex and anaemia (*P* = .008, OR 8.5; Table 4). Among the 20 patients with LVH, 10 (50%) had *PKD1* truncating mutations, 9 (45%) had *PKD1* non-truncating mutations and only 1 (5%) had a *PKD2* mutation (Table 5) (see Supplementary Tables).

**Table 3:** Association between mitral valve prolapse and truncating *PKD1* mutation.

Genotype, *n* (%)	No mitral prolapse (*n* = 67)	Mitral prolapse (*n* = 20)	Total (*N* = 87)
*PKD1* non-truncating	21 (31.3)	5 (25.0)	26 (29.9)
*PKD1* truncating	18 (26.9)	13 (65.0)	31 (35.6)
*PKD2*	28 (41.8)	2 (10.0)	30 (34.5)

**Table 4:** Association between LVH and *PKD1* mutation adjusted for hypertension, age, gender and anaemia.

Predictor	*P*-value	OR (95% CI)
Genetics (reference: *PKD2*)	.008	8.529 (1.729–42.067)
Hypertension	.334	1.886 (0.521–6.831)
Sex (reference: male)	.571	1.401 (0.436–4.504)
Age	.073	1.048 (0.996–1.104)
Anaemia	.911	0.919 (0.210–4.029)

**Table 5:** Association between LVH and *PKD1* mutations.

Genotype, *n* (%)	No LVH (*n* = 67)	LVH (*n* = 20)	Total (*N* = 87)
*PKD1* non-truncating	17 (25.4)	9 (45.0)	26 (29.9)
*PKD1* truncating	21 (31.3)	10 (50.0)	31 (35.6)
*PKD2*	29 (43.3)	1 (5.0)	30 (34.5)

## DISCUSSION

This study provides significant insights into the complex cardiovascular phenotype of ADPKD, highlighting novel genotype–phenotype correlations. We demonstrate, for the first time to our knowledge, a strong association between LVH and the presence of *PKD1* mutations, an effect independent of traditional risk factors like hypertension. Furthermore, our findings link aortic regurgitation directly to markers of renal disease severity and confirm an association between mitral valve prolapse and truncating *PKD1* mutations, adding granularity to our understanding of cardiac risk in ADPKD.

The pathogenesis of LVH in ADPKD has been debated, with uncertainty surrounding the relative contributions of hypertension versus intrinsic disease-related factors [11, 22–24]. While effective BP control, particularly with RAAS blockade, can mitigate LVH development as shown in the HALT-PKD trial (NCT00283686) [22] evidence also points towards increased LVH prevalence even in normotensive individuals [23, 24]. Our finding that *PKD1* mutations confer an ≈8.5-fold increased risk of LVH, independent of hypertension, age, sex and anaemia, strongly supports the hypothesis that intrinsic mechanisms related to polycystin-1 dysfunction directly impact myocardial structure or function. Polycystin-1 plays a role in various cellular processes, including mechanosensation, cell adhesion and calcium signalling [8]; its deficiency or dysfunction in cardiomyocytes or cardiac fibroblasts could potentially lead to aberrant growth signalling (e.g. via mammalian target of rapamycin pathways), altered calcium homeostasis, increased fibrosis or abnormal responses to mechanical stress, ultimately contributing to hypertrophy. Further mechanistic studies are warranted to elucidate these pathways.

Regarding valvular heart disease, our data confirm previous observations that the mitral valve is frequently affected in ADPKD [12–14]. Mitral regurgitation was highly prevalent (67%), although largely mild in our cohort. Interestingly, unlike a recent smaller study [15], we did not find a significant association between mitral regurgitation and genotype or renal phenotype, possibly reflecting differences in cohort size, characteristics or assessment methodology. However, we did observe a strong association between mitral valve prolapse and truncating *PKD1* mutations, aligning with findings by Lumiaho *et al.* [25]. This specific link supports the proposed mechanism involving defective primary cilia function affecting extracellular matrix integrity and leading to leaflet redundancy and prolapse [8], suggesting a direct structural consequence of severe *PKD1* mutations on valvular tissue.

Our study also uncovered a novel association between aortic regurgitation and ADPKD severity, indicated by larger renal diameters and lower eGFR. This finding likely reflects the known association between ADPKD and aortic root ectasia, which itself is linked to factors like hypertension, aging and potentially Mayo imaging class [25, 26]. Aortic root dilation is a primary cause of chronic aortic regurgitation [24, 27]. Therefore, our results suggest that aortic regurgitation in ADPKD may signify a more systemic vascular or connective tissue phenotype associated with advanced disease rather than being solely a consequence of uraemia or volume overload.

Importantly, we found no significant association between either LVH or valvulopathies and the presence of cysts in other organs (liver, pancreas, intracranial). This suggests that while originating from the same primary genetic defect, the pathways leading to cardiac abnormalities may diverge from those driving cystic development in different extrarenal sites, possibly influenced by organ-specific factors or distinct modifier genes.

This study has several strengths, including its relatively large cohort size for an ADPKD study incorporating genetic analysis, the comprehensive assessment of cardiac and systemic features and the use of multivariable models to adjust for key confounders. However, certain limitations must be acknowledged. The retrospective, single-centre design may introduce selection bias and limits generalizability. Our reliance on clinical echocardiographic reports means potential interobserver variability, and LVH was defined by interventricular septum thickness rather than the potentially more robust LV mass index, which may not have been uniformly available. The cross-sectional nature of the analysis precludes establishing causality or tracking progression over time. While we adjusted for major confounders, residual confounding by unmeasured factors remains possible. Moreover, although NGS of the GC-rich *PKD1* exon 1 can be challenging, we mitigated this limitation by achieving high overall depth (mean 118×, ≥20× in 92% of bases) and by Sanger ‘fill-in’ of any low-coverage regions, minimizing the risk of missed pathogenic variants. Finally, the sample size, while substantial overall, may have limited statistical power for detecting associations in smaller subgroups, particularly for less common valvulopathies stratified by genotype.

In conclusion, our findings underscore the importance of genotype in determining cardiovascular risk in ADPKD. The independent association of *PKD1* mutations with LVH and the link between aortic regurgitation and disease severity provide valuable prognostic information. These insights support a move towards more personalized cardiovascular risk stratification in ADPKD management. Knowing a patient’s genotype, particularly distinguishing *PKD1* from *PKD2* and identifying truncating *PKD1* mutations, could potentially guide the frequency and intensity of cardiovascular screening (echocardiography, BP monitoring) and inform therapeutic decisions. Future research should focus on prospective longitudinal studies to confirm these associations and track the progression of cardiac abnormalities in relation to genotype and renal decline. Elucidating the precise molecular mechanisms linking polycystin dysfunction with cardiac remodelling and valvulopathy is crucial for identifying potential therapeutic targets aimed at mitigating the significant cardiovascular burden of ADPKD.

## Supplementary Material

sfaf279_Supplemental_File

## Data Availability

The data underlying this article will be shared upon reasonable request to the corresponding author.
