# Supplementary material for: Cardiac complications in autosomal dominant polycystic kidney disease: links to genotype and CKD severity
Source: Clin Kidney J. 2025 Oct 1;18(11):sfaf279. doi: 10.1093/ckj/sfaf279 (PMC12585513; doi:10.1093/ckj/sfaf279)
Supplement: sfaf279_Supplemental_File [file sfaf279_supplemental_file.docx]

**SUPPLEMENTARY MATERIALS**

**Supplementary Methods:**

Genetic Analysis

***Test performed****: In silico gene panel, analysis focused on the coding regions and exon-intron junctions (±5bp) of the PKD1 (NM_001009944), PKD2 (NM_000297), HNF1B (NM_000458), and PKHD1 (NM_138694) genes. NGS sequencing performed with a Custom Bundle kit (Sophia Genetics) on a NovaSeq 6000 platform (Illumina).*

***Test characteristics****: Analytical sensitivity and specificity >99%. Mean coverage of the sequenced regions: 136.43X. For the interpretation of results, only regions with a minimum read depth of 30X were considered.*

***Confirmatory test****: NGS sequencing performed with the KAPA HyperExome Probes V2 kit (Roche) on a NovaSeq 6000 platform (Illumina).*

***Test limitations****: The presence of chromosomal regions homologous to the PKD1 gene with high sequence similarity lowers the analytical sensitivity of the test. Furthermore, due to GC-rich regions and repetitive sequences in exon 1 of the PKD1 gene, it is possible that the alignment is not perfectly reliable. The test may not identify duplications and deletions of the single exon, multiexonic, of the entire gene, complex genomic rearrangements and mutations from expansion of repeated sequences (dynamic mutations), which can be characterized with other techniques. The method has a limited resolution in the identification of mosaicisms. Due to possible GC-rich regions and repetitive sequences, the alignment with the reference sequence may not be reliable. It is possible that a variant is not identifiable due to the failure to capture the genomic region in which it is located or the lack of variant calling and variant annotation by the software used. The presence of any homologous chromosomal regions with high sequence similarity (pseudogenes) decreases the sensitivity and analytical specificity of the test. Furthermore, it is possible that a variant may not be recognized as causative of the clinical phenotype due to incomplete scientific knowledge. The interpretation of the variants was performed on the basis of the indications and clinical data provided at the time of the test request.*

***Note****: Bioinformatic analysis was performed using the BWA Aligner/DRAGEN Germline Pipeline/DRAGEN Enrichment systems. The sequences were aligned to the GRCh37 human reference genome. The Geneyx Analysis software (Knowledge-Driven NGS Analysis tool powered by the GeneCards Suite) was used for variant filtering and prioritization. The HPO, OMIM, and/or GeneReviews databases were consulted for the selection of genes associated with the clinical indication. Only variants in the selected genes with adequate read depth and quality parameters were considered*^1^*. As a rule, variants annotated on non-MANE Select transcripts (Matched Annotation from NCBI and EMBL-EBI project*^2^*; ) are not reported. The identification of a variant with a VAF (Variant Allele Frequency) <35% may be indicative of an allelic imbalance caused by a somatic event (mosaicism)*^3^*.*

*For variant filtering, the considered MAF (Minor Allele Frequency) is adjusted based on the disease prevalence. By practice, in syndromic phenotypes, variants with a MAF >0.2% are excluded from the investigation, in accordance with the definition of a rare disease established by the European Union Regulation (EC) No 141/2000. Variants were annotated according to HGVS nomenclature and classified according to standard ACMG guidelines*^4^ ^5^ *as benign (class 1), likely benign (class 2), variant of uncertain significance (VUS, class 3), likely pathogenic (class 4), or pathogenic (class 5). For the interpretation of variants, reference is made to scientific literature, the ClinVar, HGMD, and LOVD databases, and gene- and/or disease-specific databases, where available; for allele frequency, reference is made to the gnomAD v2.1.1 population database and the internal laboratory database. The nomenclature and classification of variants, particularly those of uncertain significance, may change based on updates to the reference sequence and new scientific evidence. All variants that are clinically and biologically irrelevant based on current knowledge, variants classified as benign and/or likely benign, synonymous variants, intronic variants, variants in UTR regions, or those located at non-canonical splice sites have not been reported. Typically, class 3 variants with parental segregation, found in genes associated with dominant conditions, are not reported, although the presence of incomplete penetrance or variable expressivity cannot be excluded. In trio analyses, the parents' genomic data are used exclusively for evaluating the segregation of variants present in the proband. Secondary findings in genes indicated in the current ACMG recommendations*^6^*, which are responsible for phenotypes unrelated to the clinical query, are reported only if requested for clinical exome analysis as specified on the informed consent.*

*Confirmatory testing, when performed, is carried out on a second DNA extraction and is standard practice for class 4 and 5 variants. The purpose of the test is to identify a possible cause of the patient's clinical condition and not to report any heterozygous variants associated with autosomal recessive conditions that are outside the scope of the clinical indication. We are available to perform a re-analysis of the genetic data in the event of a clinical re-evaluation of the patient. The non-identification of pathogenic variants does not exclude the possibility that the clinical indication for which the test was requested has a genetic basis. The laboratory is certified according to the UNI EN ISO 9001:2015 standard and participates in GenQA (Genomics Quality Assessment) external quality assessment schemes.*

**Supplementary References:**

1. Rehder C, Bean LJH, Bick D, et al. Next-generation sequencing for constitutional variants in the clinical laboratory, 2021 revision: a technical standard of the American College of Medical Genetics and Genomics (ACMG). *Genet Med Off J Am Coll Med Genet*. 2021;23(8):1399-1415. doi:10.1038/s41436-021-01139-4

2. Morales J, Pujar S, Loveland JE, et al. A joint NCBI and EMBL-EBI transcript set for clinical genomics and research. *Nature*. 2022;604(7905):310-315. doi:10.1038/s41586-022-04558-8

3. Avramović V, Frederiksen SD, Brkić M, Tarailo-Graovac M. Driving mosaicism: somatic variants in reference population databases and effect on variant interpretation in rare genetic disease. *Hum Genomics*. 2021;15(1):71. doi:10.1186/s40246-021-00371-y

4. Richards S, Aziz N, Bale S, et al. Standards and guidelines for the interpretation of sequence variants: a joint consensus recommendation of the American College of Medical Genetics and Genomics and the Association for Molecular Pathology. *Genet Med Off J Am Coll Med Genet*. 2015;17(5):405-424. doi:10.1038/gim.2015.30

5. Matthijs G, Souche E, Alders M, et al. Guidelines for diagnostic next-generation sequencing. *Eur J Hum Genet EJHG*. 2016;24(10):1515. doi:10.1038/ejhg.2016.63

6. Miller DT, Lee K, Abul-Husn NS, et al. ACMG SF v3.2 list for reporting of secondary findings in clinical exome and genome sequencing: A policy statement of the American College of Medical Genetics and Genomics (ACMG). *Genet Med Off J Am Coll Med Genet*. 2023;25(8):100866. doi:10.1016/j.gim.2023.100866

**Supplementary Tables:**

**Tab 1**

| **Patient** | **Genomic coordinate** | **Protein change** | **Allele frequency** | **Previous reports** | **Classification sec ACMG** |
| --- | --- | --- | --- | --- | --- |
| 1 | PKD1 NM_001009944 c.[165_171del] | p.[Leu56ArgfsTer15] | N/A | N/A | Pathogenic variant (Class 5) |
| 2 | PKD2 NM_000297  c.[2614 C>T] | p.[Arg872Ter] | N/A | ClinVar : RCV000763531  HGMD: CM994295 | Pathogenic variant (Class 5) |
| 3 | PKD2 NM_000297 c.[2046_2049delTACT] | p.[Tyr684LeufsTer3] | N/A | N/A | Likely pathogenetic variant (Class4) |
| 4 | PKD1 NM_001009944 c.[7487delC] | p.[Thr2496ArgfsTer124] | N/A | N/A | Likely pathogenetic variant (Class4) |
| 5 | PKD2 NM_000297  c.[637C>T] | p.[Arg213Ter] | 0.0000319 | ClinVar  ID: 543947  PMID: 27499327 | Pathogenic variant (Class 5) |
| 6 | PKD2 NM_000297  c.1319+1G>A | N/A | 0.00001 | Clinvar:  RCV000542204 | Pathogenic variant (Class 5) |
| 7 | PKD1 NM_001009944 c.[3268_3270delAAT] | p.[Asn1090del] | N/A | N/A | Likely pathogenic variant (Class 4) |
| 8 | PKD1 NM_001009944 c.[7546C>T] | p.[Arg2516Cys] | N/A | ClinVar:  RCV000681734  PMID: 26453610 | Likely pathogenic variant (Class 4) |
| 9 | PKD2 NM_000297  c.1256C>T | p.[Arg2516Cys] | N/A | ClinVar:  RCV005002128 | Likely pathogenic variant (Class 4) |
| 10 | PKD2 NM_000297 c.[1390C>T] | p.[Arg464Ter] | N/A | ClinVar:  ID 13521  PMID:  9402976 | Pathogenic variant (Class 5) |
| 11 | PKD2 NM_000297  c.1319+1G>A | N/A | 0.00001 | Clinvar:  RCV000542204 | Pathogenic variant (Class 5) |
| 12 | PKD2 NM_000297 c.1094+1G>A | Splicing variant | N/A | ClinVar:  RCV000379740  PMID:  22508176 | Pathogenic variant (class 5) |
| 13 | PKD2 NM_000297 c.[1449dupT] | p.[Ile484TyrfsTer42] |  | PMID:  32457805 | Pathogenic variant (Class 5) |
| 14 | PKD2 NM_000297 c.[1449dupT] | p.[Ile484TyrfsTer42] |  | PMID:  32457805 | Pathogenic variant (Class 5) |
| 15 | PKD1 NM_001009944 c.[12138+5G>A] | Single nucleotide variant | N/A | RCV005362383.1 | Variant of uncertain significance  (Class 3) |
| 16 | PKD1 NM_001009944 c.[2180T>C] | p.[Leu727Pro] | N/A | ClinVar  ID: 562302  PMID: 22383692 | Likely pathogenic variant (Class 4) |
| 17 | PKD1 NM_001009944 c.[2180T>C] | p.[Leu727Pro] | N/A | ClinVar  ID: 562302  PMID: 22383692 | Likely pathogenic variant (Class 4) |
| 18 | PKD1 NM_001009944 c.[6676G>A] | p.[Gly2226Arg] | N/A | N/A | Likely pathogenetic variant (Class 4) |
| 19 | PKD2 NM_000297 c.[1319+1G>T] | Splicing variant | N/A | N/A | Pathogenic variant (Class 5) |
| 20 | PKD1 NM_001009944 c.[1927delG] | p.[Gly644AspfsTer141] | N/A | ClinVar  ID: 803173 | Likely pathogenic variant (Class 4) |
| 21 | PKD1 NM_001009944 c.[5515T>A] | p.[Trp1839Arg] | N/A | PMID:  33964006 | Likely pathogenic variant (Class 4) |
| 22 | PKD2 NM_000297 c.[841A>G] | p.[Lys281Glu] | N/A | N/A | Variant of uncertain significance  (Class 3) |
| 23 | PKD2 NM_000297 c.[1390C>T] | p.[Arg464Ter] | N/A | ClinVar:  ID 13521  PMID: 9402976 | Pathogenic variant (Class 5) |
| 24 | PKD2 NM_000297  c.[2318A>G] | p.[His773Arg] | 0.00001 | ClinVar:  RCV002504575.2 | Variant of uncertain significance  (Class 3) |
| 25 | PKD2 NM_000297 c.[1249C>T] | p.[Arg417Ter] | 0.0000319 | Clinvar:  RCV000681683 | Pathogenic variant (Class 5) |
| 26 | PKD2 NM_000297 c.[2614C>T] | p.[Arg872Ter] | N/A | ClinVar: RCV000763531  HGMD: CM994295 | Pathogenic variant (Class 5) |
| 27 | PKD1 NM_001009944.3. c.[11402_11411dup] | p.[Ala3805SerfsTer14] | N/A | N/A | Likely pathogenetic variant |
| 28 | PKD2 NM_000297 c.[1081C>T] | p.[Arg361Ter] | N/A | ClinVar:  RCV001000981 | Pathogenic variant (Class 5) |
| 29 | PKD2 NM_000297 c.[709+1G>A] | Splicing variant | N/A | ClinVar: RCV000078584 | Pathogenic variant (Class 5) |
| 30 | PKD1 NM_001009944.3. c.1961delA | p. [Leu56ArgfsTer15] | N/A | PMID: 40507770 | Likely pathogenetic variant (class 4) |
| 31 | PKD1 NM_001009944.3  c.288-1G>T | Nonsense variant | N/A | N/A | Likely pathogenetic variant (class 4) |
| 32 | PKD1 NM_001009944.3  c.1023_1024insGG | Nonsense variant | N/A | N/A | Likely pathogenetic variant (Class 4) |
| 33 | PKD1 NM_001009944 c.[8302G>C]  c.11234G>A | p.[Val2768Leu]  p.[Gly3745Glu] | N/A  N/A | N/A  N/A | Likely pathogenic variant (Class 4)  Variant of uncertain significance ( Class 3) |
| 34 | PKD1 NM_001009944 c.[5629G>C] | p.[Ala1877Pro] | N/A | N/A | Variant of uncertain significance  (Class 3) |
| 35 | PKD1 NM_001009944 c.[4306C>T] | p.[Arg1436Ter] | N/A | ClinVar RCV000681669  PubMed: 33532864 | Pathogenic variant (Class 5) |
| 36 | PKD1 NM_001009944 c.[7741delG] | p.[Ala2581GlnfsTer29] | N/A | N/A | Likely pathogenic variant (Class 4) |
| 37 | PKD1 NM_001009944  c.9368T>A  c.11498_11512delGGCTGCGCTTCCTGC | p.[Leu3123His]  p.[Arg3833_Leu3837del] | N/A | N/A | Variants of uncertain significance  (Class 3) |
| 38 | PKD1 NM_001009944  c.4835C>T  c.9447C>G | p.[Thr1612Met]  p.[Ser3149Arg] | 0.0000842  N/A | ClinVar RCV000506637  N/A | Variants of uncertain significance  (Class 3) |
| 39 | PKD1 NM_001009944 c.[6360C>G] | p.[Tyr2120Ter] | N/A | N/A | Pathogenic variant (class 5) |
| 40 | PKD1 NM_001009944 c.[11834C>G] | p.[Thr3945Arg] | N/A | N/A | Variant of uncertain significance  (Class 3) |
| 41 | PKD1 NM_001009944 c.[1295C>T] | p.[Ala432Val] | N/A | ClinVar:  ID: 636617  PMID: 32816041 | Pathogenic variant (class 5) |
| 42 | PKD2 NM_000297 c.[916C>T] | p.[Arg306Ter] | 0.00000398 | ClinVar  RCV000449565  PMID: 33532864 | Pathogenic variant (class 5) |
| 43 | PKD1 NM_001009944 c.[445C>T] | p.[Gln149Terl] | N/A | ClinVar:  RCV003231682.1 | Pathogenic variant (class 5) |
| 44 | PKD1 NM_001009944 c.[12503dupG] | p.[Ser4169Leufs41] | N/A | PMID:29038287 | Pathogenic variant (class 5) |
| 45 | PKD1 NM_001009944 c.[4521G>A] | p.[Trp1507Ter] | N/A | HGMD: CM203198  doi: 10.1016/j.kint.2019.08.038. | Pathogenic variant (class 5) |
| 46 | PKD1 NM_001009944 c.[6778_6780delATT] | p.[Ile2260del] | N/A | ClinVar:  RCV002284778.15 | Likely pathogenetic variant |
| 47 | PKD1 NM_001009944 c.[5014_5015delAG] | p.[Arg1672Glyfs98] | N/A | ClinVar:  RCV000989457.28PMID:  22383692 | Pathogenic variant (class 5) |
| 48 | PKD1 NM_001009944 c.[12175C>T] | p.[Gln4059Ter] | N/A | N/A | Pathogenic variant (class 5) |
| 49 | PKD1 NM_001009944 c.[11693_11702dupCGCTGCCTCT] | p.[Leu3902AlafsTer62] | N/A | N/A | Probably pathogenic variant (class 4) |
| 50 | PKD2 NM_000297 c.[2614C>T] | p.[Arg872Ter] | N/A | ClinVar: RCV000763531  HGMD: CM994295 | Pathogenic variant (class 5) |
| 51 | PKD2 NM_000297 c.[1158T>A] | p.[ Tyr386Ter] | N/A | PMID:29338003  PMID: 26467025 | Pathogenic variant (class 5) |
| 52 | PKD2 NM_000297  c. [1624C>T] | p. (Gln542Ter) | N/A | N/A | Likely pathogenetic variant (Class 4) |
| 53 | PKD1 NM_001009944 c.[11935C>T] | p.[Gln3979Ter] | N/A | PMID:22508176 | Pathogenic variant (class 5) |
| 54 | PKD1 NM_001009944 c.[3485_3492del] | p.Aspl162clyfsTer46 | N/A | N/A | Likely pathogenetic variant (Class 4) |
| 55 | PKD2 NM_000297 c.[1827delA] | p.[Ala610ArgfsTer64] | N/A | N/A | Likely pathogenic variant (class 4) |
| 56 | PKD1 NM_001009944 c.[3032_3056del] | p.[Val1011GlyfsTer19] | N/A | N/A | Likely pathogenic variant (class 4) |
| 57 | PKD1 NM_001009944 c.[755dup] | p.[Pro253AlafsTer8] | 0.0000145 | ClinVar  ID: 562290 | Likely pathogenic variant (class 4) |
| 58 | PKD1 NM_001009944  c.4362_4366delinsTGAGTCATT | p.[Asn.1455GlufsTer69] | N/A | N/A | Likely pathogenic variant (class 4) |
| 59 | PKD2 NM_000297  c.[2614 C>T] | p.[Arg872Ter] | N/A | ClinVar : RCV000763531  HGMD: CM994295 | Pathogenic variant (class 5) |
| 60 | PKD1 NM_001009944 c.[10084C>T] | p.[Gln3362Ter] | N/A | ClinVar:  RCV000712571.3 | Pathogenetic variant (class 5) |
| 61 | PKD1 NM_001009944 c.[275A>G] | p.[Tyr2092Cys] | N/A | PMID: 12842373 | Probably pathogenic variant (class 4) |
| 62 | PKD1 NM_001009944 c.[9608delG] | p.[Asp3204ThrfsTer112] | N/A | N/A | Probably pathogenic variant (class 4) |
| 63 | PKD1 NM_001009944  c.[7394T>C] | p.[Leu2465Pro] | N/A | N/A | Variant of uncertain significance  (Class 3) |
| 64 | PKD1 NM_001009944 c.[9608delG] | p.[Asp3204ThrfsTer112] | N/A | N/A | Probably pathogenic variant (class 4) |
| 65 | PKD2 NM_000297 c.[9608delG] | p.[Asp3204ThrfsTer112] | N/A | N/A | Probably pathogenic variant (class 4) |
| 66 | PKD1 NM_001009944 c.[8311G>A] | P[Glu2771Lys]] | N/A | ClinVar:  RCV000763371.23 | Pathogenetic variant (class 5) |
| 67 | PKD2 NM_000297 c.[2614C>T] | p.[Arg872Ter] | N/A | ClinVar: RCV000763531  HGMD: CM994295 | Pathogenic variant (Class 5) |
| 68 | PKD1 NM_001009944 c.[6384C>G] | p.[Asn2128Lys] | N/A | ClinVar:  1676237 | Likely pathogenetic variant |
| 69 | PKD2 NM_000297 c.[916C>T] | p.[Arg306Ter] | 0.00000398 | ClinVar  ID: 397507  PMID: 33532864 | Pathogenic variant (class 5) |
| 70 | PKD1 NM_001009944  c.[11180_11181delinsAT] | p.[Met3727Asn] | N/A | N/A | Variant of uncertain significance  (Class 3) |
| 71 | PKD1 NM_001009944  c.[4041_4042delCA] | p. [His1347Glnfs*83] | N/A | ClinVar:  RCV001292032.2  ClinVar:  RCV002499521.3 | Pathogenetic variant (class 5) |
| 72 | PKD1 NM_001009944  c.[4041_4042delCA] | p. [His1347Glnfs*83] | N/A | ClinVar:  RCV001292032.2  ClinVar:  RCV002499521.3 | Pathogenetic variant (class 5) |
| 73 | PKD1 NM_001009944 c.[11884C>T] | p.[Gln3962Ter] | N/A | ClinVar:  RCV001292123).  PMID: 30989420 | Pathogenic variant (class 5) |
| 74 | PKD1 NM_001009944  c.4835C>T  c.9447C>G | p.[Thr1612Met]  p.[Ser3149Arg] | 0.0000842  N/A | ClinVar RCV000506637  N/A | Variants of uncertain significance  (Class 3) |
| 75 | PKD2 NM_000297 c.[1319+1G>A] | Splicing variant | 0.00000798 | ClinVar  RCV000496001  PMID: 22863389 | Pathogenic variant (class 5) |
| 76 | PKD1 NM_001009944 c.[4041_4042delCA] | P.[His 1347Glnfs*83]] | N/A | ClinVar:  RCV001292123).  PMID: 30989420 | Pathogenic variant (class 5) |
| 77 | PKD2 NM_000297 c.[1319+1G>T] | Splicing variant | N/A | N/A | Pathogenetic variant (class 5) |
| 78 | PKD1 NM_001009944 c.[11693_11702dupCGCTGCCTCT] | p.[Leu3902AlafsTer62] | N/A | N/A | Probably pathogenic variant (class 4) |
| 79 | PKD1 NM_001009944 c.[9387_9391delCCGGG] | p.[Arg3130LeufsTer47] | N/A | N/A | Probably pathogenic variant (class 4) |
| 80 | PKD1 NM_001009944 c.[4041_4042delCA]  c.[8653C>T] | p.[His1347Glnfs*83]  p.[Arg2885Trp] | N/A | ClinVar:  RCV001292032.2  ClinVar:  RCV002499521.3 | Pathogenic variant (class 5) |
| 81 | PKD2 NM_000297 c.[2614 C>T] | p.[Arg.872Ter] | 0.00000398 | CilnVar:  ID 448036  PMID:  10541293 | Pathogenic variant (class 5) |
| 82 | PKD2 NM_000297 c.[1449dupT] | p.Ile484TyrfsTe42r | N/A | N/A | Pathogenetic variant (class 5) |
| 83 | PKD1 probe 14094-L24670 x1 | Microdel esone 22 | N/A | N/A | Likely pathogemetic variant (class 4) |
| 84 | PKD1 NM_001009944 c.[9625C>T] | p.[Arg3209Cys] | 0.0000109 | N/A | Variants of uncertain significance  (Class 3) |
| 85 | PKD2 NM_000297 c.[1081C>T] | p.[Arg361Ter] | N/A | ClinVar:  RCV001000981 | Pathogenic variant (Class 5) |
| 86 | PKD2 NM_000297 c.[1094+1del] | Splicing variant | N/A | ClinVar:  ID 1699919 | Pathogenetic variant (class 5) |
| 87 | PKD2 NM_000297 c.[327insG] | p.[Met110Asnfs103*] | N/A | N/A | Likely pathogemetic variant (class 4) |

**Tab 2 (a-b)**

**Associations of aortic regurgitation with average bilateral renal diameters and eGFR: multivariate models**

| **Predictor** | **P Value** | **OR (95% C.I. for OR)** |
| --- | --- | --- |
| AVERAGE BILATERAL RENAL DIAMETERS  SEX (Reference: male)  AGE | 0.360  0.720  0.001 | 1.048 (0.948-1.158)  2.279 (0.930-5.584)  1.069 (1.028-1.112) |

**b**

| **Predictor** | **P Value** | **OR (95% C.I. for OR)** |
| --- | --- | --- |
| eGFR  SEX (reference: male)  AGE | 0.078  0.061  0.078 | 0.981 (0.961 1.002)  2.337 (0.962 5.677)  1.044 (0.995 1.095) |

**Tab 3 (a-n)**

**a Univariate association between aortic regurgitation and aneurisms**

| **Predictor** | **P Value** | **OR (95% C.I. for OR)** |
| --- | --- | --- |
| Aneurisms | 0.496 | 0.479 (0.059-3.981) |

**b Univariate association between aortic regurgitation and extrarenal cysts**

| **Predictor** | **P Value** | **OR (95% C.I. for OR)** |
| --- | --- | --- |
| Extrarenal cysts | 0.617 | 0.778 (.0291-2.080) |

**c Univariate association between mitral prolapse and aneurisms**

| **Predictor** | **P Value** | **OR (95% C.I. for OR)** |
| --- | --- | --- |
| Aneurisms | 0.439 | 1.766 (0.418-7.455) |

**d Univariate association between mitral prolapse and extrarenal cysts**

| **Predictor** | **P Value** | **OR (95% C.I. for OR)** |
| --- | --- | --- |
| Extrarenal cysts | 0.632 | 1.238 (0.517-2.967) |

**e Univariate association between mitral regurgitation and aneurisms**

| **Predictor** | **P Value** | **OR (95% C.I. for OR)** |
| --- | --- | --- |
| Aneurisms | 0.141 | 0.360 (0.092-1.404) |

**f Univariate association between mitral regurgitation and extrarenal cysts**

| **Predictor** | **P Value** | **OR (95% C.I. for OR)** |
| --- | --- | --- |
| Extrarenal cysts | 0.334 | 1.519 (0.651-3.542) |

**g Univariate association between tricuspid regurgitation and aneurisms**

| **Predictor** | **P Value** | **OR (95% C.I. for OR)** |
| --- | --- | --- |
| Aneurisms | 0.918 | 1.074 (0.277-4.161) |

**h Univariate association between tricuspid regurgitation and extrarenal cysts**

| **Predictor** | **P Value** | **OR (95% C.I. for OR)** |
| --- | --- | --- |
| Extrarenal cysts | 0.958 | 1.021 (0.479-2.174) |

**i Univariate association between pulmonary regurgitation and aneurisms**

| **Predictor** | **P Value** | **OR (95% C.I. for OR)** |
| --- | --- | --- |
| Aneurisms | 0.999 | 0.000 (0.000 |

**l Univariate association between pulmonary regurgitation and extrarenal cysts**

| **Predictor** | **P Value** | **OR (95% C.I. for OR)** |
| --- | --- | --- |
| Extrarenal cysts | 0.406 | 0.408 (0.049-3.380) |

**m Univariate association between LVH and aneurisms**

| **Predictor** | **P Value** | **OR (95% C.I. for OR)** |
| --- | --- | --- |
| Aneurisms | 0.815 | 1.186 (0.284-4.961) |

**n Univariate association between LVH and extrarenal cysts**

| **Predictor** | **P Value** | **OR (95% C.I. for OR)** |
| --- | --- | --- |
| Extrarenal cysts | 0.067 | 0.409 (0.157-1.065) |

**Tab 4 Association between mitral valve prolapse and truncating *PKD1* mutation.**

| **Comparison** | **Odd Ratio** | **p-value** | **Adjusted p-value** |
| --- | --- | --- | --- |
| PKD1 Non-Truncating **vs** PKD1 Truncating | 3.033 | 0.089 | 0.178 |
| PKD1 Non-Truncating **vs**  PKD2 | 0.300 | 0.231 | 1.231 |
| PKD1 Truncating **vs**  PKD2 | 0.099 | 0.002 | **0.007** |

Pairwise Fisher's Exact Test Results. Correction method: Holm-Bonferroni.

**Tab 5 Association between mitral valve prolapse and *PKD1* truncating mutation adjusted for hypertension age, gender and anemia.**

| **Predictor** | **P Value** | **OR (95% C.I. for OR)** |
| --- | --- | --- |
| *GENETICS (Reference: PKD2)*  GENETICS (*PKD1* truncating)  GENETICS (PKD2)  HYPERTENSION  SEX (reference: male)  AGE  ANEMIA | **0.037**  0.219  0.319  0.100  0.269  0.146 | 3.950 (1.085 14.373)  0.328 (0.055 1.942)  1.885 (0.542 6.560)  2.761 (0.822 9.274)  1.027 (0.979 1.078)  0.340 (0.079 1.455) |

**Tab 6 Comparison of interventricular septal thickness in *PKD1* truncating, *PKD1* non-truncating and *PKD2* genotypes**

| **Group 1** | **Group 2** | **Mean diff** | **P-adj** | **Lower** | **Upper** |
| --- | --- | --- | --- | --- | --- |
| PKD1 non-truncating | PKD1 truncating | 0.067 | 0.989 | 1.098 | 1.232 |
| PKD1 non-truncating | PKD2 | 1.251 | **0.034** | 2.425 | 0.077 |
| PKD1 truncating | PKD2 | 1.318 | **0.017** | 2.441 | 0.196 |

Tukey's HSD Post-Hoc Test Results.*PKD1* non-truncating **vs** *PKD2* adjusted p value: 0.034; *PKD1* truncating **vs** *PKD2* adjusted p value: 0.017.

**Tab 7 Association between left ventricular hypertrophy and *PKD1* mutation.**

| **Comparison** | **Odd Ratio** | **p-value** | **Adjusted p-value** |
| --- | --- | --- | --- |
| PKD1 non-truncating **vs** PKD1 truncating | 0.899 | 1.00000 | 1.00 |
| PKD1 non-truncating **vs**  PKD2 | 0.065 | 0.00363 | **0.011** |
| PKD1 truncating **vs**  PKD2 | 0.072 | 0.00574 | **0.011** |

Pairwise Fisher's Exact Test Results. Correction method: Holm-Bonferroni. *PKD1* non-truncating **vs** *PKD2* adjusted p value= 0.011; *PKD1* truncating **vs** *PKD2* adjusted p value= 0.011
